# Cellular basis of learning and memory in the carotid body

**DOI:** 10.3389/fnsyn.2022.902319

**Published:** 2022-08-15

**Authors:** Olivia M. S. Gold, Emma N. Bardsley, Anna P. Ponnampalam, Audrys G. Pauza, Julian F. R. Paton

**Affiliations:** Manaaki Manawa - The Center for Heart Research, Department of Physiology, Faculty of Health and Medical Sciences, University of Auckland, Auckland, New Zealand

**Keywords:** glutamate, GABA, synaptic plasticity, long term potentiation, type I cells, type II cells

## Abstract

The carotid body is the primary peripheral chemoreceptor in the body, and critical for respiration and cardiovascular adjustments during hypoxia. Yet considerable evidence now implicates the carotid body as a multimodal sensor, mediating the chemoreflexes of a wide range of physiological responses, including pH, temperature, and acidosis as well as hormonal, glucose and immune regulation. How does the carotid body detect and initiate appropriate physiological responses for these diverse stimuli? The answer to this may lie in the structure of the carotid body itself. We suggest that at an organ-level the carotid body is comparable to a miniature brain with compartmentalized discrete regions of clustered glomus cells defined by their neurotransmitter expression and receptor profiles, and with connectivity to defined reflex arcs that play a key role in initiating distinct physiological responses, similar in many ways to a switchboard that connects specific inputs to selective outputs. Similarly, within the central nervous system, specific physiological outcomes are co-ordinated, through signaling via distinct neuronal connectivity. As with the brain, we propose that highly organized cellular connectivity is critical for mediating co-ordinated outputs from the carotid body to a given stimulus. Moreover, it appears that the rudimentary components for synaptic plasticity, and learning and memory are conserved in the carotid body including the presence of glutamate and GABAergic systems, where evidence pinpoints that pathophysiology of common diseases of the carotid body may be linked to deviations in these processes. Several decades of research have contributed to our understanding of the central nervous system in health and disease, and we discuss that understanding the key processes involved in neuronal dysfunction and synaptic activity may be translated to the carotid body, offering new insights and avenues for therapeutic innovation.

## Introduction

The link between structure and function is pervasive throughout biology and is observed at the microscale, from the structure of DNA to amino acids and folding proteins, through to the macroscale structure of organs and entire organisms. Given that biological structure is so fundamentally related to function, we suggest that the carotid body is morphologically structured in a way that enables the detection, integration, and responses to various stimuli. In this manner, we hypothesize that the carotid body is like the brain, acting as a switchboard for signal integration, where numerous incoming signals are simultaneously received, processed in parallel and translated into a range of appropriate physiological outputs (Table 1). In this review, we discuss evidence suggesting that the partitioned discrete regions of clustered cells within the carotid body respond to specific stimuli characterized by their expression of different neurotransmitters, peptides, and receptors. Yet, the orchestration of co-ordinated and synchronous physiological responses implies that these discrete compartments within the carotid body are inter-connected. Thus, we propose that the carotid body is built upon a network of cells and connections, where the structural and spatial proximity of these elements influences the response dynamics of the carotid body in a manner reminiscent of the brain where regions and nuclei are linked to their defined physiological outcomes. Indeed, the cell types and their roles within the carotid body share many similarities to the neurons and astrocytes of the brain, including their responses to signals in the form of electrical excitability, vesicular-mediated neurotransmitter release with an appropriate cadence for postsynaptic activation, mechanisms for signal termination, and the assistance of a network of supporting cells (López-Barneo et al., 2016). Other functional similarities include the expression of components for synaptic plasticity within the carotid body, including the presence of glutamate and gamma-aminobutyric acid (GABA) systems (Oomori et al., 1994; Li et al., 2020), the most common excitatory and inhibitory neurotransmitters of the nervous system, respectively. We suggest that the pathophysiology of the carotid body, contributing to diseases such as hypertension and diabetes, could be linked to deviations in these processes. Therefore, recognizing ubiquitous hallmarks of cellular dysfunction such as those seen in neurodegenerative diseases may be relevant to pathologies of the carotid body, offering new avenues for therapeutic innovation. We start this review by contrasting the carotid body with the brain and then describe the presence of glutamate and GABA systems in the carotid body and their similarities to the central nervous system (CNS).

**TABLE 1:** Summary of carotid body responses to a diverse range of stimuli.

Stimuli	Effect	References
Hypoxia, hypercapnia/low pH	Excitation	Lahiri et al., 2006
Insulin	Excitation	Conde et al., 2014
Leptin	Excitation	Kim et al., 2021
High and low glucose	Excitation	Gao et al., 2014
lysophosphatidic acid	Excitation	Jendzjowsky et al., 2021
Glucagon like 1 peptide	Inhibition	Pauza et al., 2022
Dopamine	Inhibition	Pijacka et al., 2016
Temperature	Excitation	Baron and Eyzaguirre, 1977
Adrenaline/noradrenaline	Excitation	Peng et al., 2014
Sympathetic nerves	Excitation	Felippe et al., 2022
Inflammation	Excitation	Iturriaga et al., 2022
Aldosterone	Excitation	Lincevicius., personal communication

## Structural similarities between the carotid body and the brain

### Organ-level structural specializations support functional outputs

The human brain is a highly complex organ composed of anatomically distinct and heterogeneous regions that are dynamically interconnected by thousands of neural links, that span across multiple levels of organization; from the scale of single neurons and their synapses to distinct brain regions and their interconnecting circuitry (Sporns, 2011). The topological architecture of the brain is critical for enabling partitioned responses within different regions, yet also supports and enables the integration of parallel responses from different regions, providing a mechanism for diverse yet synchronous, co-ordinated, and physiologically appropriate outputs (Sporns et al., 2005). The carotid body is most recognized for its acute responses to hypoxia, yet a wide range of diverse stimuli from intrinsic and extrinsic sources can activate the carotid body, resulting in a number of different physiological outputs. We propose that the structure and topological organization of the carotid body enables the detection and processing of numerous and diverse stimuli, to facilitate co-ordinated, appropriate, and parallel reflex responses.

The carotid body is a small ovoid-shaped bilateral organ, located at the bifurcation of the common carotid artery. It is believed to be the primary sensor of blood gases in the body and is key to the coupling of ventilation and cardiovascular systems ensuring adequate oxygenation of organs. For instance, the chemoreflex is commonly initiated after detecting a low oxygen concentration in the blood. Through a number of putative mechanisms this results in modulation of potassium channels in glomus cells causing their depolarisation and release of transmitters that depolarises the terminals of petrosal neurones that send projections into the brainstem. This results in activation of the chemoreflex arc which acts on the ventral respiratory column in the medulla oblongata causing an increase in ventilation (Koshiya et al., 1993); several respiratory neuron types project to the RVLM (Koshiya and Guyenet, 1994) to cause enhanced respiratory modulation of vasomotor sympathetic activity causing blood pressure to rise (Moraes et al., 2011), and to the cardiac and laryngeal projecting parasympathetic pre-ganglionic neurons in the nucleus ambiguus causing bradycardia and bronchoconstriction respectively (Simms et al., 2007; Moraes et al., 2021).

Additional circulatory specializations within the carotid body such as specialized sinuses provide an intra-organ mechanism by which specific stimuli are channeled to distinct glomus cell clusters (Lübbers et al., 1977; Brognara et al., 2021). Intriguingly, it appears that arterioles within the carotid body are located predominantly to one side, making the ‘vascular side’ more sensitive to sympathetic innervation (I Felippe & JFR Paton – unpublished observation), highlighting the importance of the spatial arrangement of the carotid body. Efferent parasympathetic fibers are also found to innervate the carotid body, arising in the petrosal ganglia, paraganglia within glossopharyngeal and carotid sinus nerve tracts, and possibly within the carotid body itself (Campanucci and Nurse, 2007). Afferent signals from the sensory petrosal ganglia may also participate in retrograde carotid body activity (McDonald and Mitchell, 1975).

**FIGURE 1:**
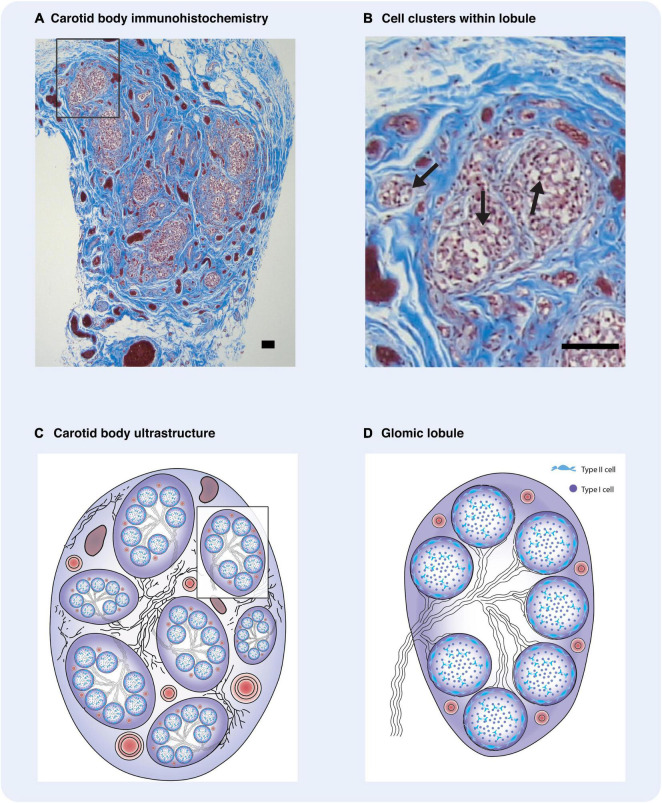
Carotid body ultrastructure is highly organized. **(A)** Immunohistochemistry of human carotid body with Masson Trichrome stain depicting clusters of type I cells. **(B)** Carotid body regional cluster of type I cells. Often type I cells are arranged in smaller clusters within each lobule. **(A,B)** were provided to us by Dr. Rexson Tse from ADHB. **(C)** Diagrammatic representation of the carotid body and its constituents. Regional clusters or lobules are defined. Between the lobules are numerous bundles of myelinated nerves, glomic arteries, and veins, embedded in a fibrous capsule. **(D)** Diagrammatic illustration of a glomic lobule. The cluster is composed of cellular clusters, with a central core of type I cells surrounded by type II cells. Some myelinated nerves enter the lobules and branch between the clusters. These defined regions are supplied by intra-lobular glomic arterioles which terminate as capillaries adjacent to type I cells. **(C,D)** adapted and reproduced with permission from Heath and Smith (2012). Arrows – type I glomus cells. Scale bars –20 μm.

**FIGURE 2:**
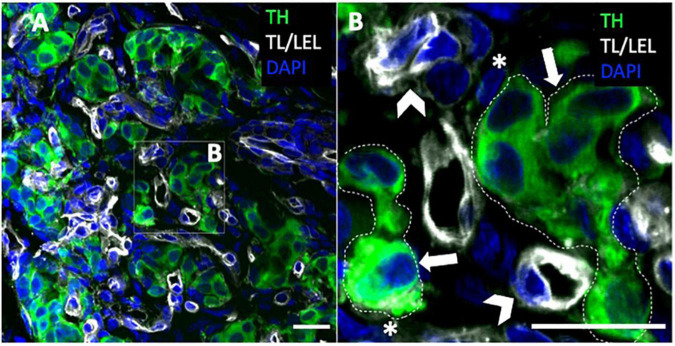
Carotid body type I and type II cells. **(A,B)** Immunohistochemistry of rat carotid body with glomus cell marker (TH) depicting clusters of type I cells. Blood vessels are associated with tomato lectin/lycopersicon esculentum lectin (TL/LEL) signal. Magnified panel selected to highlight the type II cells and type I cell clusters. Arrowheads – blood vessels; asterisk – type II cell; thick arrows – type I cell. Scale bar –20 μm. DAPI indicates 4’,6-diamidino-2-phenylindole; and TH, tyrosine hydroxylase.

**FIGURE 3:**
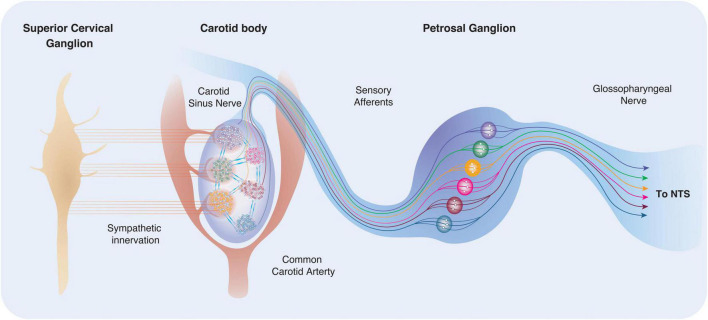
Carotid body location, innervation and connectivity. The carotid bodies reside in the bifurcation of the common carotid artery bilaterally and are highly vascularised. Recent work suggests that arterioles within the carotid body are located predominantly to one side, making the “vascular side” more sensitive to sympathetic innervation. Type I cells receive postganglionic sympathetic fibers from the SCG via two nerves: the GGN and the carotid sinus nerve (CSN). Within the carotid body, regional clusters may be sensitive to distinct and specific stimuli (for example through differences in exposure, or differences in phenotype). Activation of different lobules or clusters may be connected to defined physiological outputs and reflex arcs. This theory has previously been described as the “Ribbon Cable” (e.g., a multi-wire planar cable) hypothesis (Zera et al., 2019).

A cross-section of the carotid body reveals that large groups of type I and type II cells are assembled in highly vascularised regional clusters, like tightly woven hubs; and we suggest that these regions act as distinct subunits of the organ itself (Figure 1). Within rat carotid body regional clusters, type I cells are small and numerous populations of heterogenous (McDonald and Mitchell, 1975), electrically active cells, with distinct neurotransmitter and receptor characteristics, which are encapsulated by many type II cell processes (Ross, 1959) (Figure 2). The petrosal afferent sensory neurons that relay the type I cell signals to the brain are also phenotypically heterogenous, and different populations can also be defined by the receptors they possess (Zera et al., 2019). We hypothesis that there are subsets of phenotypically diverse type I cells that respond to specific and distinct stimuli, evoking the release of a discrete set of neurotransmitters onto directly appending petrosal afferent terminals, and thus triggering the activation of appropriate physiological response to the stimulus; this forms the basis for the Ribbon Cable Hypothesis (Zera et al., 2019) (Figure 3). There is a paucity of evidence to support our hypothesis, however, Pijacka et al. (2016) has shown that P2X3 receptor antagonism within the carotid body attenuates the sympathetic reflex response sparing any effect on the phrenic nerve response in the spontaneously hypertensive rat (SHR). For these physiological responses to be co-ordinated there must be a method for communication between the discrete regions of the carotid body. Understanding this connectivity may help us to expand our knowledge of signal interpretation and output in the healthy, developing, aging and diseased carotid body (Figure 3).

## Cellular and molecular similarities between the carotid body and the brain

### Type I cells and neurons

#### Cellular and molecular characteristics

**FIGURE 4:**
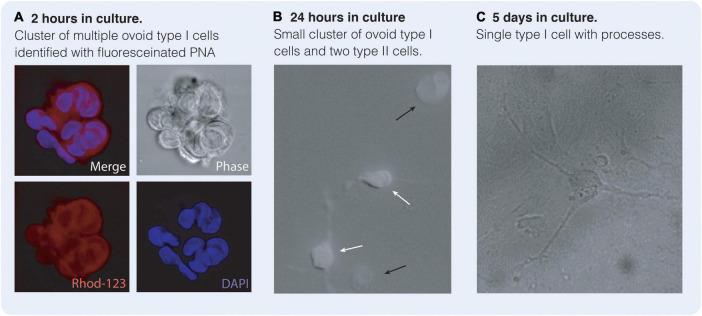
Type I and type II cells in culture reminiscent of central nervous system (CNS) neurones and astrocytes. Carotid body type I cells may be identified by their affinity for Peanut Agglutinin (PNA) when labeled with a fluorescent marker such as Rhodamine-123 (Rhod-123). **(A)** Cluster of ovoid type I cells after 2 h in culture identified with PNA-labeled Rhod-123 and DAPI. **(B)** After 24 h in culture type I cells (black arrow) are still ovoid in shape. Type II cells (white arrows) are present with some processes and are astrocytic in nature. Type II cells are not PNA positive. **(C)** After 5 days in culture, type I cells remain PNA positive but have grown multiple processes appearing substantially more neuronal in phenotype. (Bardsley E and Buckler K, unpublished data).

Type I cells are derived from the neural crest (Le Liévre and Le Douarin, 1975) with a neuronal lineage derived from the superior cervical ganglion (Pardal et al., 2007; Porzionato et al., 2008; Navarro-Guerrero et al., 2016). They are the most abundant cell type in the carotid body with the cell number ranging between 12,000 type I cells in the rat and up to 60,000 in the cat (Kumar and Prabhakar, 2012). Electron microscopic studies have identified two classes of cells within the rat carotid body: type I glomus cells and type II sustentacular cells (McDonald and Mitchell, 1975; Duchen et al., 1988). Glomus cells (8–20 μm diameter) have an oval or polygonal shape with a spherical or ovoid nucleus and a well-developed Golgi apparatus and two types of vesicles: clear smooth and coated vesicles with the latter having electron-dense material indicating the presence of catecholamines (Duchen et al., 1988) (Figure 2). Type-II cells are distinguished from glomus cells by the absence of both cytoplasmic organelles and dense-core vesicles (Duchen et al., 1988). Type I cells are a heterogenous population comprising type A and B, whereby, type A glomus cells are spherical in shape with diameters ranging between approximately 8–15 μm, where type B glomus cells appear more irregular in shape (Ortega-Sáenz et al., 2013). Type I glomus cells (particularly the B subtype) possess cytoplasmic processes, some more than 40 μm in length (McDonald and Mitchell, 1975) which increase in length with age (Donnelly, 1995). When type I cells are cultured *in vitro*, these processes tend to disappear initially and the cells become more spherical, however in cell cultures maintained for over 48 h their processes reappear (Kumar and Prabhakar, 2012) (Figure 4). Regardless of these phenotypical differences, type I cells may be distinguished from type II cells and other cell types by their affinity to peanut agglutinin (PNA) that may be labeled with a fluorescent marker (Kim et al., 2009).

Most traditional neurotransmitters have been identified within the carotid body, including adenosine triphosphate (ATP), adenosine, acetylcholine (ACh), dopamine, noradrenaline, serotonin (5-HT), angiotensin II and histamine. A range of neuropeptides and neuromodulators have also been observed including substance P and neuropeptide Y (Kumar and Prabhakar, 2012), while ATP is recognized as the primary excitatory transmitter for hypoxia (Alcayaga et al., 2000). Light microscope studies on the carotid bodies from several species has identified at least two types of glomus cells (historically termed A and B) that contain different size and abundance of dense-core vesicles (McDonald and Mitchell, 1975). Whether they can be further sub-classified (e.g., by their transmitter and peptide reactivities) remains to be determined, however these differences in phenotype support the notion that type I cells are a heterogenous population where differences in cellular morphology as well as vesicle contents may be linked to different sensitivities to stimulants, connections and functions.

Type I cells are a class of electrically excitable cells that depolarise when exposed to stimuli and have been described as “*essentially neuron-like in [their] properties*” (Duchen et al., 1988). Neurons of the CNS are also a widely heterogeneous population that are physically rather distinct to type I cells particularly with respect to size, with the majority of neuronal soma ranging between 20 to 70 μm (Lingley et al., 2018). Like type I cells, neurons depolarise to stimuli. The resting potential of acutely isolated rat type I cells is generally between –40 to –60 mV (Kumar and Prabhakar, 2012; Buckler, 2015), slightly higher than the reported –70 mV usually recorded from neurons of the CNS (Williams and Marshall, 1987; Disterhoft et al., 1988; Lynch and Barry, 1989; Brown et al., 1993), yet similar to the reported mean resting potential of –58 mV for superior cervical ganglion neurons (Lamas et al., 2002). Resting membrane potential is maintained through the balance of inward (most likely Na^+^), outward (most likely K^+^) currents and the activity of the Na^+^/K^+^ ATPase pump (Buckler, 2015). It is widely accepted that exposure to depolarising stimuli, if of sufficient magnitude evokes an all-or-nothing electrical event, promoting Ca^2+^ entry via voltage-gated Ca^2+^ channels, and exocytosis of neurotransmitter-containing vesicles. In the carotid body this results in excitation of petrosal afferent nerve terminals (Donnelly, 1995; Kumar and Prabhakar, 2012). Yet the upstream events that promote depolarisation of the type I cells, including the identity of the oxygen sensor and the contribution of the various ion channels expressed by glomus cells remains much less clear. The initial depolarisation of type I cells has been linked to the closure of selective background K^+^ channels in the rat, rabbit, cat and mouse (Kumar and Prabhakar, 2012), and although the precise channels involved in depolarisation are not entirely resolved, there appears to be a clear role for the Twik-related acid-sensitive K^+^ channel (TASK) family in maintaining rat type I cell resting potential, where the initial membrane depolarisation to hypoxia appears to be a consequence of TASK-1 / TASK-3 channel inhibition (Buckler, 2007, 2015). In neurons, the initial depolarisation is driven by the activation of voltage-gated Na^+^ channels (Wang et al., 2017). In type I cells, there appears to be a species-dependent difference with regards to type I cell currents involved in the initiation of depolarisation. For example, voltage-gated Na^+^ channels play a role in the sensory potentials of rabbit type I cells (Buckler, 2015), whereas in rat this early stage of depolarisation is also linked to Na^+^ ion influx but the current is most likely carried via a voltage insensitive, non-selective cation channel (Carpenter and Peers, 2001; Buckler, 2015). Intriguingly, the genetic components of voltage-gated Na^+^ channel expression are present in type I cells in rat, although the functional relevance of this is not clear (Caceres et al., 2007). As Fieber and McCleskey have pointed out, however, this discrepancy in voltage-gated Na^+^ channel activity is also observed in other organs, yet it appears to have minimal impact on overall cellular activity (Fieber and McCleskey, 1993).

Regardless of the ion channels involved in depolarisation, both cell types encounter an increase in intracellular Ca^2+^ that triggers the intracellular mobilization of neurotransmitter-containing vesicles to the plasma membrane, and the release of the vesicular contents into the extracellular synaptic space. This chemical signal evokes an electrical cascade in the postsynaptic cell until the signal is terminated via enzymatic breakdown of the transmitter, reuptake by neighboring cells, and/or the activation of presynaptic autoreceptors terminating vesicular release. Regarding intercellular signal transduction, both type I cells of the carotid body and neurons of the CNS are tightly connected to adjacent cells by several classes of gap junction, enabling intracellular Ca^2+^ (and other signaling molecules) to spread to cellular neighbors. In the carotid body, there is immunohistochemical evidence for the expression of type I cell connexin molecules including connexin-32 (Cx32), Cx36 and Cx43 (Abudara et al., 1999; Kondo, 2002; Frinchi et al., 2013) between adjacent type I cells, type I and type II cells (Kondo, 2002), and occasionally between glomus cells and nerve terminals (Kondo, 2002). Whether gap junctions between type I cell and petrosal afferent are functional and relevant to chemoreceptor transduction is not known. It has been accepted that the petrosal nerve endings are not chemo-sensitive themselves and depend on the release of neurotransmitters from type I glomus cells for the generation of post-synaptic potentials. However, although chemical transmission between type I cells and the afferent nerve ending appears to be necessary, evidence suggests it may not be sufficient for the entire transduction process (Eyzaguirre, 2007). Thus, functional evidence for electrical coupling has been identified in the carotid body. In the brain, neuronal connections via gap junctions promote synchronization of neural oscillations (Pernelle et al., 2018). The contribution of gap junctions to chemoreceptor transmission within the carotid body is not fully resolved but may facilitate the synchronization of electrical activity within glomus cell clusters to stimuli (Abudara et al., 1999) as well as amplify afferent output. As with neurones in the brain, the carotid body also possess supporting cells and these are discussed below.

### Type II cells and astrocytes

The type II sustentacular cells of the carotid body (Figure 2B) form networks of supporting cells. They are derived from mesenchymal cells and are of glial cell lineage (Kameda, 2020). Aside from their striking visual similarity to astrocytes and other glial cells of the brain, type II cells contain intermediate size filaments and express glial markers including glial fibrillary acidic protein (GFAP) vimentin, and S100 protein (Kameda, 2020). In healthy carotid bodies, the number of type I cells outnumbers the type II cells by a factor of approximately three to five (McDonald and Mitchell, 1975; Verna, 1979; Hansen, 1985). This ratio is relevant to the structural arrangement of type I and type II cells within their clusters, where between three to five adjacent type I cells are encompassed by the thin cytoplasmic process of a single type II cell (McDonald and Mitchell, 1975; Verna, 1979). While type II cells are not electrically excitable by membrane depolarisation, they do display Ca^2+^ signals in response to neurotransmitter stimulation (Duchen et al., 1988; Nurse et al., 2018). A primary role for astrocytes in the CNS is signal modulation, where the precise presynaptic and postsynaptic neuronal response to stimulation is unequivocally tied to the activity of its neighboring astrocytes (Parpura and Haydon, 2000; Horvat et al., 2021). From a morphological standpoint, astrocytic processes interact intimately with neurons, forming tripartite synapses (Kettenmann et al., 1996; Haydon, 2001) and close connections with up to 100,000 neuronal terminals depending on the brain region (Südhof, 2018); as well as interacting with endothelial cells of capillaries [Figure 2; Haydon (2001)]. Astrocytes and glial cells of the CNS exhibit a purinergic, glutamatergic, and serotonergic neurochemical profile, and are both a source and a responder to these chemical transmitters (Chung et al., 2015). Stimulation of these cells raises intracellular Ca^2+^ signals which is propagated via gap junctions to neighboring astrocytes in a wave-like manner, albeit on a time scale that is six orders of magnitude slower than neuronal action potentials (Haydon, 2001). The subsequent release of transmitters enables the amplification of neuronal signals and the synchronization of cellular networks (Lee et al., 2008). Astrocytic ATP is thought to be released in a Ca^2+^-independent manner and may be responsible for long-distance signaling via release from distant processes, whereas glutamate is Ca^2+^-dependent and released locally onto neuronal terminals, providing an extensive network of seemingly fine-point control (Haydon, 2001). A similar phenomenon is observed in the carotid body, where type II cells express a wide array of functional receptors and form tripartite-like synaptic connections with type I cells and blood vessels [(Leonard et al., 2018); see Figure 2] providing a mechanism by which type II cells can modulate the type I responses to hypoxia, controlling local and distant signals to modulate the sensory output of the carotid body in a co-ordinated manner. Whether type II cells control local blood flow by coupling it with the metabolic demand of type I cells is unknown. Alternatively, and hypothetically, it may be that type II cells act to augment information transfer from capillaries to type I cells in the process of stimuli detection. Thus, in an analogous way to glial cells, we may think of type II cells as a central integrative hub bridging, co-ordinating and amplifying communications between groups of excitable (and non-excitable) cells.

Astrocytic processes are extensive and provide a responsive scaffold to support and enable the refining of neural connectivity, synaptic distribution, and circuit architecture throughout the brain (Shao et al., 2013; Molofsky et al., 2014; Chung et al., 2015; Stogsdill and Eroglu, 2017). In the rodent brain, most excitatory synapses form around the second to third week after birth, occurring alongside the differentiation and maturation of astrocytes (Chung et al., 2015). Although synapses can form in the absence of astrocytic processes, the presence of astrocytes and glia control the location, strength, and survival of synapse formation (Chung et al., 2015; Stogsdill and Eroglu, 2017). These mechanisms are evident in both vertebrates and invertebrates, and experiments in Caenorhabditis elegans (C. elegans) suggest that the relationship between astrocytes and synapse formation is conserved (Shao et al., 2013; Stogsdill and Eroglu, 2017). Given the close spatial proximity between type I cells, petrosal afferents, and type II cells in the carotid body (Leonard et al., 2018) and the similarities with astrocytes of the CNS, it is tempting to speculate that type II cells may play developmental and supporting roles for carotid body synapses in a similar manner. We propose that much like the brain, type II cells provide a foundation upon which the structure, organization, and connectivity of the carotid body is built, to aid and support synapse construction and circuit formation within the carotid body early in development (Bénard and Hobert, 2009; Molofsky et al., 2014; Stogsdill and Eroglu, 2017) as well as maintaining the carotid body architecture throughout life. One aspect of synaptic formation and maintenance in the brain involve neurotrophic factors, which are also found in abundance in the carotid body.

### Neurotrophic factors

An abundant number of growth factors are present in the CNS including glial-derived neurotrophic factor (GDNF) which promotes neuronal survival (Pöyhönen et al., 2019) and brain-derived neurotrophic factor (BDNF) that has been linked to synapse formation (Parkhurst et al., 2013), dendritic growth, action potential generation, synaptic activity (Galati et al., 2016), and plasticity (Miranda et al., 2019). Neuronal growth factor (NGF) and neurotropin-3 (NT-3), among others, are also widely expressed in the brain (Parkhurst et al., 2013; Pöyhönen et al., 2019). Although our understanding of their function islimited, the carotid body has been found to be strongly immunoreactive for several neurotrophic factors (Stocco et al., 2020). Type I cells in the carotid body are strongly immunoreactive for GDNF (Villadiego et al., 2005; Izal-Azcárate et al., 2008; De Caro et al., 2013) which exerts a trophic effect on chemoafferent nerves of the petrosal ganglion (Porzionato et al., 2008). NT-3 is also present (Hertzberg et al., 1994; Atanasova and Lazarov, 2014) as are NGF and its cognate receptor Tropomyosin receptor kinase A (TrkA) (Hertzberg et al., 1994; Yamamoto and Iseki, 2003), which have been associated with carotid body hyperplasia in some cases (Porzionato et al., 2008). At least 90% of type I cells also possess high levels of BDNF (Hertzberg et al., 1994; Izal-Azcárate et al., 2008) that is directly linked to the growth and survival of tyrosine hydroxylase (TH)-positive petrosal chemoafferent nerves (Hertzberg et al., 1994). In the CNS, BDNF plays a role in enhancing spontaneous firing, increasing mEPSP amplitude and frequency through the activation of the TrkB receptor (Galati et al., 2016), and can play a role in maintaining synaptic connectivity and plasticity (Parkhurst et al., 2013; Stogsdill and Eroglu, 2017). Might BDNF play similar roles to these in the carotid body? Evidence suggests that a major stimulus for BDNF release is coupled to neuronal activity and specifically, activation of the purinergic P2X4 receptor by ATP (Parkhurst et al., 2013). In the carotid body, ATP is released from type I cells in response to hypoxia (Buttigieg and Nurse, 2004; Masson et al., 2008), so it is plausible that BDNF is also released in the carotid body at active synaptic sites, modulating transmission. Moreover, altered levels of BDNF, GDNF, NGF and NT-3 have been associated with a range of diseases associated with cellular dysfunction and neurodegeneration including Alzheimer’s disease, Parkinson’s disease, Amyotrophic Lateral Sclerosis, Multiple Sclerosis, and ischaemia during stroke (Villadiego et al., 2005; Miranda et al., 2019; Pöyhönen et al., 2019). Given the importance of neurotrophin signaling for cellular survival and synaptic activity it is likely that the optimal concentration of local neurotrophic factors is acutely regulated, thus a role for altered trophic factor signaling in carotid body pathology such as sleep apnoea, hypertension or other diseases associated with altered firing patterns is plausible (Porzionato et al., 2008).

## Transplanting carotid body into the brain

The fact that the carotid body has been transplanted into the brain supports the hypothesis that the former is similar to the latter. Indeed, the similarities such as the tyrosine hydroxylase phenotype of carotid body, the structure and function of type I and II cells with neurons and astrocytes of the CNS have not gone unnoticed. This has led to transplantation of the carotid body into specific brain regions as a strategy to ameliorate symptoms of some neurodegenerative diseases. Autotransplantation of carotid bodies into the striatum of clinical patients and animal models with Parkinson’s disease (PD) has resulted in some intriguing measures of clinical success (Minguez-Castellanos et al., 2007). The rationale was based on the similar properties of type I cells, and their ability to produce catecholamines and neurotrophins to aid and replace the degenerating striatal neurons. Given that type I cells can exhibit a dopaminergic profile, it was thought that autotransplantation might ameliorate some of the motor symptoms of PD (Minguez-Castellanos et al., 2007). Moreover, several other neurotransmitter systems are also affected in PD including noradrenaline, 5-HT, ACh, ATP and adenosine (Burnstock et al., 2011). Functionally, type I cells may possess an ability to receive and integrate different modality inputs and given that they are electrically excitable release transmitters with an appropriate cadence onto postsynaptic terminals. However, whether integration of different stimuli occurs at the level of a single glomus cell and release of transmitter type in a stimulus specific manner remains to be elucidated. Both the preclinical and clinical studies showed several measures of improvements including the longevity of the carotid body transplant in animal models of PD, where the graft survived for the duration of the animals’ lifespan (>70% survival; (Minguez-Castellanos et al., 2007) and retained a healthy cellular phenotype (Toledo-Aral et al., 2003; Porzionato et al., 2008). This was based on the presence of tyrosine hydroxylase in the transplanted tissue. The grafted glomus cells were found to extend outside of the graft forming neurite processes and catecholaminergic synapses with embedded striatal neurons. Importantly, some amelioration of the motor symptoms was observed in animals and patients, as well as some regeneration and sprouting of striatal neurons (Espejo et al., 1998). These improvements reflected the ability of the graft to integrate and produce sufficient levels of dopamine and other neurotransmitters to supplement the degenerative striatum. In addition, neurotrophic factors (particularly GDNF; see above) were released from the carotid body graft into the milieu conferring a neuroprotective and neurorestorative effect on host nigrostriatal neurons, promoting survival of the graft, surrounding tissue and connectivity within it (Toledo-Aral et al., 2003; Minguez-Castellanos et al., 2007; De Caro et al., 2013). The carotid bodies’ resistance to hypoxic environments may have enabled the transplant to survive unlike previous attempts using other tissue types such as mesencephalic neurons (Toledo-Aral et al., 2003; Minguez-Castellanos et al., 2007) and adrenal chromaffin cells (Dunnett and Björklund, 1999). Unfortunately, from a clinical standpoint, the magnitude and duration of pathological improvement was found to be moderate, as improvements depreciated over time due to the progressive nature of the disease. Yet despite this, the authors reported that the transplant showed “*true biological effects derived from carotid body grafting*” that were *“mediated by carotid body cells*” in the striatum (Minguez-Castellanos et al., 2007). We feel that the similarities between the carotid body and brain are strongly supported by these transplantation trials. We now raise the question of whether glutamatergic and GABAergic mechanisms so commonly found in the brain also exist in the carotid body.

## The ubiquitous nature of glutamate and gamma-aminobutyric acid (GABA)

### Synapse and circuit development

Glutamate and GABA, major modulators of excitatory and inhibitory signals in the adult mammalian brain, are abundant during embryonic development and play a critical role in nervous structure and network establishment (Luján et al., 2005; Zhou and Danbolt, 2014). Specifically, both glutamatergic and GABAergic signaling are involved in the regulation of neuronal proliferation, migration, and differentiation, as well as formation of synaptic contacts, neural plasticity and the refinement of neuronal circuits later in life (Behuet et al., 2019), where estimates suggest that around 90% of synapses in the adult mammalian brain utilize glutamate (Andersen et al., 2021). The evolutionary relevance and importance of glutamate in synaptic formation, development and plasticity is emphasized by experiments involving *Drosophila melanogaster* and *C. Elegans*, where these functions are conserved (Petersen et al., 1997; Featherstone et al., 2005; Bozorgmehr et al., 2013; Lau et al., 2013; Aponte-Santiago et al., 2020; Bai and Suzuki, 2020). Also conserved is GABAergic signaling that provides inhibitory inputs to neural circuits, suggesting that these mechanisms emerged early in evolution (Das et al., 2011; Behuet et al., 2019; Bai and Suzuki, 2020; Cuentas-Condori et al., 2020). If the carotid body is architected with organized networks of cells forming circuit-like arrangements that are responsive to experience, then, given the importance of glutamate and GABA in synapse and circuit formation in the brain, coupled with evidence of the existence of both glutamate and GABA systems from published RNAseq data (Pauza et al., 2022), we will explore evidence for the existence of glutamate and GABA in the carotid body.

### Glutamate and gamma-aminobutyric acid (GABA) in the carotid body

**FIGURE 5:**
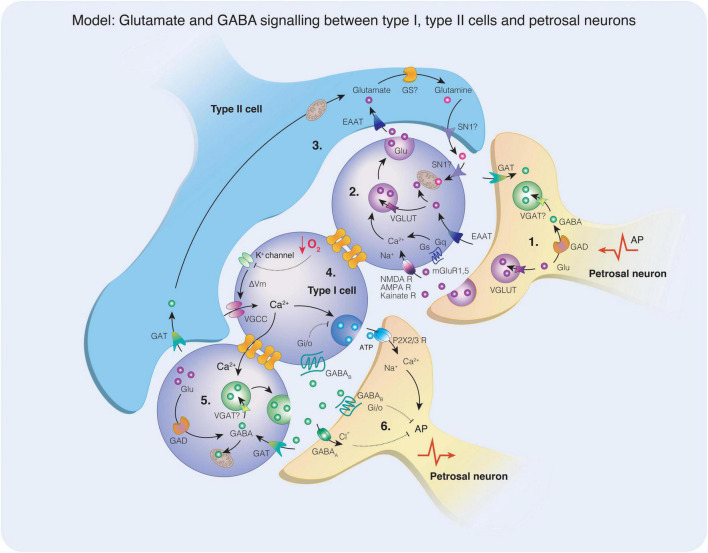
A model depicting glutamate and gamma-aminobutyric acid (GABA) signaling mechanisms in the carotid body. (1) Glutamate is packaged into synaptic vesicles via the VGLUT transporter or may be converted to GABA via the activity of glutamic acid decarboxylase (GAD). The presence of vesicular GABA transporter (VGAT) required for the uptake of GABA into synaptic vesicles has not yet been identified in petrosal neurons. A retrograde action potential (AP) may stimulate the release of glutamate and / or GABA from petrosal neuron terminals into the synaptic space. (2) Glutamate acts on the ionotropic glutamate receptors (NMDA, AMPA, Kainate) or the metabotropic glutamate receptors (mGluR1, mGluR5) on type I cells. The activation of the ionotropic receptors does not appear to affect the acute response to hypoxia, however in chronic intermittent hypoxia (CIH), NMDA receptor subunits and AMPA receptor subunits were upregulated. Activation of NMDA receptors in CIH elevates the hypoxic response, perhaps due to increased membrane expression. Vesicular glutamate transporter (VGLUT) is also expressed in type I cells and may facilitate the uptake of glutamate into synaptic vesicles for release. Increases in intracellular Ca^2+^ triggers vesicle mobilization and glutamate release and spreads to adjacent neurons via gap junctions. (3) Excitatory amino acid transporters located on type I or type II cells remove glutamate from the synaptic cleft, terminating the action of glutamate on membrane receptors. In type II cells, glutamate may be converted to glutamine via the activity of glutamine synthase (GS), although this enzyme has not yet been observed in the carotid body. Glutamine may be released from type II cells via the glutamine transporter (SN1) and taken up via the same transporter into type I cells where it can enter mitochondria and the TCA cycle as a metabolite or be converted for glutamate for use as a transmitter. (4) When a type I cell is exposed to hypoxia, K^+^ channels close raising membrane potential that opens voltage-gated Ca^2+^ channels. An influx of Ca^2+^ triggers the release of synaptic vesicles, such as ATP in this example. Synaptic ATP acts on postsynaptic petrosal neuron P2X2/3 receptors resulting in Na^+^ and Ca^2+^ influx that contribute to AP generation. (5) Intracellular Ca^2+^ flows via gap junctions to an adjacent type I cell, stimulating the release of GABA. The synthesis of GABA may occur in type I cells through the conversion of glutamate via the activity of GAD. GABA may be taken up by mitochondria and enter the TCA cycle as a metabolite or transported into synaptic vesicles via the action of VGAT, although the presence of VGAT has not yet been identified in type I cells. (6) Synaptic GABA may act on GABA_B_ receptors coupled to Gα_i/o_ G proteins localized on type I cells or petrosal neurons. Alternatively, GABA may act on GABA_A_ ionotropic receptors facilitating Cl^–^ entry and hyperpolarising the postsynaptic membrane. As indicated the inwardly directed current, depolarization, and increased frequency discharge (Iturriaga et al., 2007; Zhang et al., 2009) indicate an excitatory action of GABA on PG neurons. Activation of both subtypes of GABA receptor typically oppose AP generation. Reuptake of GABA occurs via the GABA transporter (GAT) terminating the GABA signal.

**FIGURE 6:**
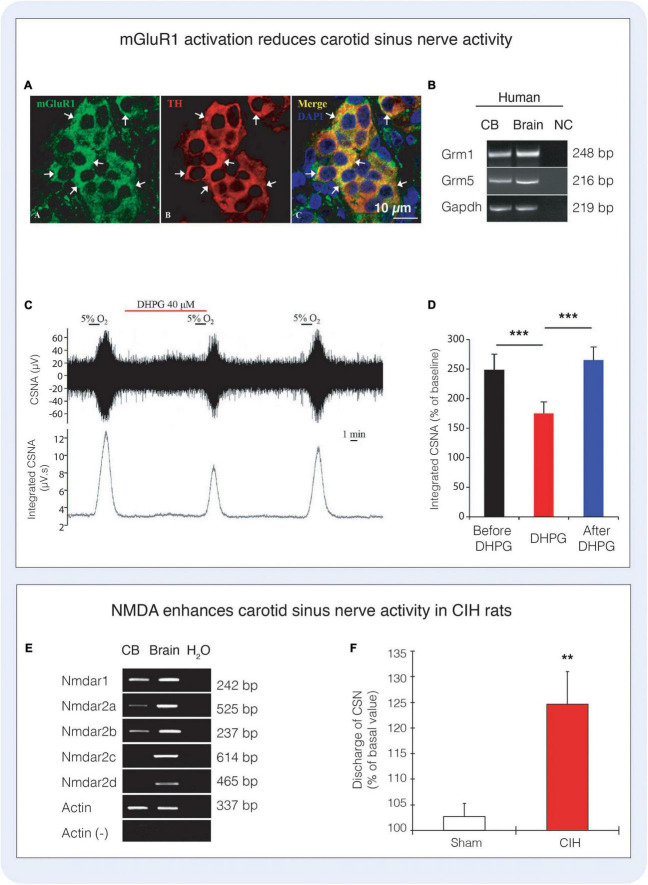
Glutamate receptors and signaling in the carotid body. **(A)** Immunofluorescence depicts co-localisation of metabotropic glutamate receptor type 1 with tyrosine hydroxylase (TH) expressing type I cells in the rat carotid body. **(B)** RT-PCR highlights the expression of mRNA encoding mGluR1 and mGluR5 (*Grm1*, *Grm5*) in the human carotid body. **(C,D)** Activation of mGluR1 with the agonist 3,5-dihydroxyphenylglycine (DHPG) significantly reduces carotid sinus nerve activity (CSNA) in rat. Figures **(A–D)** reproduced with permission from Li et al. (2021). **(E)** The NMDA receptor subunits *Nmdar1, Nmdar2a, Nmdar2b* are present in the rat carotid body. **(F)** Administration of 40 μM NMDA significantly increases carotid sinus nerve activity in rats exposed to chronic intermittent hypoxia (CIH), however NMDA did not alter the response to acute hypoxia (not shown). Figures **(E,F)** reproduced with permission from Liu et al. (2009b).

Glutamate immunoreactivity was first demonstrated in the carotid body nearly two decades ago, however, at the time it was considered to be present as a metabolite rather than have a role as a transmitter (Torrealba et al., 1996). More recently, evidence of glutamatergic neurotransmission has come to light, including the presence of the vesicular glutamate transporters (VGLUT) in the carotid body of rat and human, where VGLUT3 has been shown to be localized on TH-positive type I glomus cells (Liu et al., 2018; Li et al., 2020) and VGLUT2 on P2X3-receptor expressing petrosal nerve afferents, particularly terminals appending type I cells (Yokoyama et al., 2014) (Figures 5, 6). These findings are consistent with the storage of glutamate in synaptic vesicles (Wallén-Mackenzie et al., 2010). Equally important was the observation that the excitatory amino acid transporters (EAAT 2 and 3) are present in the carotid bodies of human and rat (Li et al., 2020). Expression has been found to be localized to type I and II cells suggesting that both cell types may be involved in the reuptake of glutamate and clearance from the synapse. Whether type II cells are involved in the conversion of glutamate to glutamine for its return to type I cells in a mechanism reflective of the glutamate-glutamine shuttle found in the brain (Andersen et al., 2021) remains to be shown, but the presence of type II localized EAATs certainly supports this prediction. Additional evidence supporting a role for glutamate as a neurotransmitter in the carotid body, includes the presence of the ionotropic N-Methyl-D-aspartate (NMDA) receptor subunits (*Nmdar1, Nmdar2a, Nmdar2b*), α-amino-3-hydroxy-5-methyl-4-isoxazolepropionic acid (AMPA) receptor subunits (*Gria1,2,3*), and Kainate receptor subunits (*Grik2, Grik5*) (Liu et al., 2018) as well as elements of the postsynaptic density (*Psd95, Psd93, Sap97*) (Liu et al., 2009b; Liu et al., 2018). The expression of the metabotropic glutamate receptor type 1 (*Grm, mGluR1*) was also shown to be expressed in the rat and human carotid body (Li et al., 2021) with the distribution of these glutamate receptors localized to type I glomus cells (Figures 5, 6). However, there is a lack of action of elevated concentrations of sodium glutamate (42 mM) on the responses of carotid bodies *in vitro* (Baron and Eyzaguirre, 1977; Alcayaga et al., 1988; Iturriaga et al., 1991).

**FIGURE 7:**
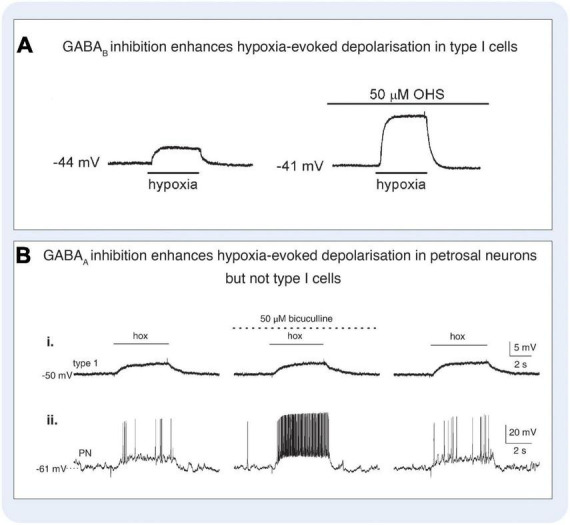
Gamma-aminobutyric acid (GABA) modulated signaling in the carotid body. **(A)** GABA_B_ antagonist hydroxysaclofen (OHS) increases hypoxia-evoked depolarisation in rat type I cells. Under control conditions (left panel), hypoxia induced a depolarisation which was increased in the presence of 50 μM OHS (right panel). Current-clamp recordings were made from a type I cell in a cluster. Application of hypoxia (PO_2_, 5 mmHg) is indicated by the horizontal bars below each trace. Figure reproduced with permission from Fearon et al. (2003). **(B)** GABA_A_ antagonist bicuculline increases hypoxia-evoked depolarisation in rat petrosal neurons but not type I cells. Simultaneous gramicidin perforated-patch recordings were obtained from a type I cell (that was part of a cluster, and an adjacent petrosal neuron (PN). Depolarizing responses to hypoxia (hox; PO_2_, 5 mmHg) were recorded in both the type I cell (i) and the functional PN (ii), where suprathreshold responses were evoked. In the presence of the 50 μM bicuculline, there was a marked potentiation of the PN response resulting in a robust increase in spike discharge (ii, middle trace), whereas the type I cell response was unaltered (i, middle trace). Reversibility of these responses following wash-out of the drug is shown (right traces). Figure reproduced with permission from Zhang et al. (2009).

Components of the GABAergic signaling system are also prevalent in the carotid body (Figure 7) and the sensory neurons of the petrosal ganglion, providing a physiological inhibitory counterpart to glutamate’s excitatory inputs. Indeed, GABA is present within glomus cells (Oomori et al., 1994; Fearon et al., 2003) and in petrosal neuron cell bodies and neural fibers (Igarashi et al., 2009), as is glutamate decarboxylase (GAD) implicating both cell types in the conversion of glutamate to GABA (Zhang et al., 2009). Regarding GABAergic transmission, mRNAs of the ionotropic GABA_A_ receptor subunits *including α1, α2, β2, γ2S, γ2L* and *γ3* (excluding δ) were found in petrosal neurons, with GABA_A_ receptors shown to be localized to sensory nerve endings directly apposing TH-positive type I cells. At present, experimental data indicates that type I cells might not express functional GABA_A_ receptors, however, metabotropic GABA_B_ receptor subunits (GABA_B1_ and GABA_B2_) appear functional on type I cells (Fearon et al., 2003). Several GABA transporters have also been found in both glomus cells and petrosal neurons, suggesting that both cell types are involved in the clearance of GABA from the synapse (Zhang et al., 2009) (Figure 5).

Thus, the expression of all the necessary molecular components involved in glutamate and GABAergic signaling are strongly indicative of a functional neurotransmitter system within the carotid body (Oomori et al., 1994; Igarashi et al., 2009; Li et al., 2021). Whether glutamate and GABA are involved in synapse development and the structural architecture of the carotid body is another intriguing possibility. However, the conserved nature of glutamate and GABA in these fundamental processes certainly supports this proposal, providing a new avenue for investigation.

### Chemoreflex modulation by glutamate and gamma-aminobutyric acid (GABA)

The presence of functional markers of glutamatergic and GABAergic signaling in the carotid body has led researchers to investigate a role for these transmitters in the chemoreflex response to hypoxia. A role for glutamate in chemoreflex modulation is not entirely clear. Li et al. (2021) found that the activation of mGluR1 inhibits the carotid sinus nerve response to acute hypoxia by almost 30% whereas inhibition of mGluR1 increased the carotid sinus nerve response to a similar degree (Li et al., 2021). However, the mechanisms underpinning the role of mGluR1 in translating oxygen sensing processes to chemotransduction are far from established. Liu et al. (2009b) demonstrated that rats exposed to CIH increased the expression of functional NMDA glutamate receptors suggesting that glutamatergic signaling may be involved in chemoreflex plasticity of the CB in response to CIH (Liu et al., 2009b). There is currently no functional evidence of AMPA receptor signaling in the carotid body, thus, an avenue to explore. Experiments involving the role of inhibitory GABAergic signaling on responses to acute hypoxia are also limited, however inhibition of GABA_B_ receptors has been shown to enhance the hypoxia-evoked activation of type I cells (Figure 7) and petrosal neurons. Moreover, application of GABA_B_ antagonists in the absence of a hypoxic stimulus facilitated membrane depolarisation in type I cells, and induced spike activation in neurons adjacent to type I cell clusters, consistent with a tonic endogenous inhibitory role for GABA in the carotid body (Fearon et al., 2003). In another rat study, Zhang et al. (2009) showed that bicuculline (50 μM) had no effect on type I cell resting membrane potential or responses to hypoxia, but increased depolarization and spiking responses in PG neurons, highlighting some differences in experimental paradigms. Nevertheless, both research groups identified the local, tonic synthesis of GABA in the carotid body and the inhibitory effect of GABA on glomus cells directly, where the cellular mechanisms were shown to be linked to the opening of background K^+^ channels, specifically the activation of a TASK-1-like conductance (Fearon et al., 2003). Thus, the effects of GABA on the carotid body appear to raise the threshold for type I cell activation suggesting hyperpolarisation of their membrane potential, suppressing the release of excitatory neurotransmitters (Fearon et al., 2003). The co-expression of background K^+^ channels and GABA_B_ receptors is prevalent in numerous neuronal cell types (Misgeld et al., 1995), raising the possibility that this may be a common mechanism for the control of cellular excitability. Regarding ionotropic-mediated GABA signaling, GABA_A_ receptors are predominantly expressed on petrosal neurons with limited expression within the carotid body itself (Zhang et al., 2009). At concentrations sufficient to induce sedation, the benzodiazepines diazepam and midazolam have been shown to partially inhibit the hypoxic ventilatory response in cats, rats, and rabbits, supposedly through positive allosteric modulation of GABA_A_ (Gueye et al., 2002; Kim et al., 2006; Igarashi et al., 2009), although the effects on ventilation in humans appear to be mixed (Alexander and Gross, 1987; Dahan and Ward, 1991; Mak et al., 1993). Recent work has shown that when petrosal neurons are exposed to both an excitatory stimulus such as ATP combined with GABA, the excitatory response is blunted indicative of the hyperpolarising effects of GABA. Zhang et al. (2009) (Figure 7) showed that petrosal ganglion neurons are depolarized by GABA, in a dose-dependent manner, inducing an inwardly directed current that reversed near –40 mV, effect blocked by bicuculline (a GABA_A_ receptor antagonist). Similar results were shown by Iturriaga et al. (2007). Thus, in basal conditions GABA depolarizes petrosal ganglion neurons. Additionally, Iturriaga et al. (2007) showed that petrosal ganglion neurons connected to the carotid sinus nerve increase their discharge frequency in response to GABA (Figure 5), suggesting a depolarizing action of GABA on petrosal ganglion neurons. These experiments highlight the possibility that upon release from type-I cells GABA excites the chemoreflex reflex arc and auto-inhibits at the same time. Whether specific ‘inhibitory’ and ‘excitatory’ glomus cells exist remains to be validated but such a finding would further support carotid body-brain analogies. Clearly, more work is required to fully understand the role of these small signaling amino acids in the context of carotid body function including oxygen sensing and signaling transduction.

### Cellular plasticity in the carotid body

Neural plasticity is the capacity of the nervous system to modify itself functionally and structurally and to adapt to repeated demands, using past experience to shape a response (von Bernhardi et al., 2017; Südhof, 2018). In this regard, diseases such as sleep apnoea expose the carotid body to several repeated periods of intermittent hypoxia. From a systems perspective, this has been shown to result in enhanced chemoreflex responses that underpin physiological changes such as sympathetic overactivity, which is a key prognostic indicator for morbidity and mortality associated with hypertension and heart disease and is a significant risk factor for negative cardiovascular events (Peng et al., 2003; Sforza and Roche, 2016). From a functional standpoint, changes to the carotid body in obstructive sleep apnoea include enhanced responsiveness of type I cells and increased petrosal nerve firing following a hypoxic challenge (Peng et al., 2003). This exaggerated response known as long term facilitation reflects an adaptation of the type I-petrosal pathway in a manner reminiscent of neural plasticity although the cellular mechanisms that underpin it are far from established. In the CNS, glutamate signaling particularly via AMPA, and NMDA receptor activation plays a significant role in several forms of neuronal plasticity (Lisman, 2017). Given the presence of glutamate and these receptors in the carotid body, it raises the question as to the involvement of glutamate-induced plasticity, not only during disease, but for learning and memory that may be important for setting the magnitude of chemoreflex evoked physiological responses.

Recent evidence has shown that NMDA receptor activation stimulates and enhances carotid sinus nerve activity (Figure 6) in a rat model of chronic intermittent hypoxia (CIH), whereas inhibition of the NMDA receptor reduces carotid sinus nerve responses to endothelin-1 activation, which is itself upregulated in CIH (Rey et al., 2006; Liu et al., 2009b). Thus, blockade of carotid body NMDA receptor signaling appears to attenuate the plastic changes that occur following CIH and effectively ameliorates the enhanced chemoreflex that is associated with system pathology. These functional changes in CIH also correspond with changes in the expression of key components related to glutamatergic signaling in the carotid body, including the upregulation of the mRNA and protein encoding the NMDA receptor subunits (*Nmdar1, Nmdar2a, Nmdar2b*) (Liu et al., 2009b) and the AMPA receptor subunit GluA1 (*Gria1*) (Liu et al., 2018). Moreover, in the same model of CIH, mRNA encoding the amino acid transporter *EAAT3* was also found to be upregulated relative to controls, whereas *VGLUT3* and *EAAT2* were downregulated, supporting the theory that glutamate transporters are dynamic and expression can be modulated upon requirement (Gonçalves-Ribeiro et al., 2019). Naturally, changes in gene expression may occur as primary events that negatively contribute to, or cause, cellular pathology; or alternatively, may arise secondarily as a compensatory preventative mechanism, for example to mitigate against elevated glutamate and subsequent cellular toxicity. Whether glutamate facilitates maladaptive plastic alterations in diseases associated with enhanced chemoreflex activity such as hypertension is an intriguing possibility. Thus, the notion is advanced that sympathetic overactivity itself shows hallmarks of neuronal plasticity and pathway enhancement, including an increased responsiveness to stimulation (Bardsley et al., 2018b; Herring et al., 2019; Bardsley and Paterson, 2020). Our previous RNA-seq data also supports this idea, where the results highlighted a significant role for altered glutamate signaling in the sympathetic stellate ganglia of the SHR. Key changes were seen in the transcripts encoding the glutamate receptors including AMPA receptor subunits (*Gria1-3*), NMDA subunit (*Grin2b*), Kainate receptor subunits (*Grik1,2*) and metabotropic glutamate receptor 7 (*Grm7*) among others (Bardsley et al., 2018a), mirroring similar changes to those reported in the carotid body in CIH. Further work will be required to establish whether these changes in mRNA expression translate to functional differences in glutamate signaling in disease; however, given the evidence we feel that the notion that glutamatergic signaling may play a role in altered plasticity in the carotid body in disease is worthy of discussion and may open therapeutic doors in the future.

## An imbalance in synaptic excitation and inhibition

A finely tuned balance between excitatory and inhibitory transmission is crucial for appropriate signaling and normal neural circuit function, and mounting evidence has implicated neural hyperactivity (Busche et al., 2008) as an early-stage functional hallmark of disease. Chronic elevations in synaptic glutamate, and subsequent excitotoxicity plays a critical role in the pathophysiology of many neurodegenerative diseases of the CNS including PD, Alzheimer’s disease, Amyotrophic Lateral Sclerosis, Huntington’s diseases, Schizophrenias, and other major psychiatric disorders (Ambrosi et al., 2014; Plitman et al., 2014; Lewerenz and Maher, 2015; Iovino et al., 2020). Reduced inhibitory GABAergic signaling has recently been implicated in many of these same diseases highlighting the importance of an excitatory / inhibitory balance for appropriate neuronal function (Walker et al., 1984; Plitman et al., 2014; Li et al., 2016; Kim et al., 2017; Kiernan et al., 2019; Xu et al., 2020).

**FIGURE 8:**
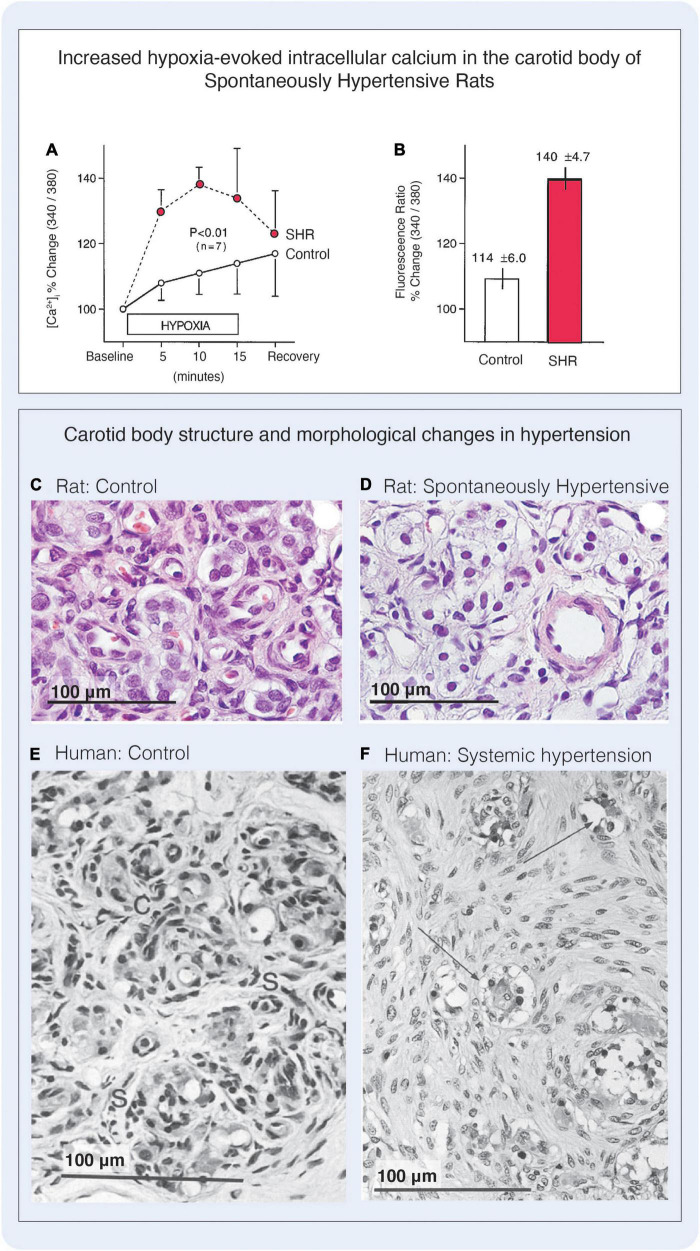
Excitability and cellular changes in the carotid body in hypertension. **(A)** Hypoxia-induced intracellular Ca^2+^ is elevated in type I cells of SHR compared with levels measured in normotensive rats (‘Control’). Cells were loaded with Fura-2 and intracellular Ca^2+^ was measured via sequential changes in the fluorimetric ratio (340/380 nm) during 15-min exposure to hypoxic superfusion (PO_2_, 55 mmHg). Simultaneous paired hypoxic exposure shows greater hypoxic responses in carotid bodies of the SHR strain compared with the F-344 rat. *n* = number of rats. **(B)** Peak Ca^2+^ responses to hypoxia were greater in SHR carotid bodies compared with F-344 rats. Figures A and B reproduced with permission from Weil et al. (1998). **(C,D)** Haematoxylin and Eosin (H&E) stain showing carotid body structure in control normotensive rats and L-NAME hypertensive rats. The L-NAME hypertensive model was found to have an enlarged carotid body, that was due to enhanced extracellular matrix deposition. The density of type I cell nuclei was decreased by 31% in the L-NAME group, although the type I cells themselves appeared hypertrophied and the cytoplasm contained vacuoles. **(E,F)** H&E stains from human carotid bodies. **(E)** Depicts carotid body lobule from a woman of 71 years. The glomic tissue is arranged into discrete clusters each comprising a central core of rounded type I cells (labeled C for chief cell) surrounded by a thin, discontinuous rim of elongated type II cells (labeled S for sustentacular). **(F)** Depicts carotid body of a 61-year-old man with coarctation of the aorta. In this congenital disorder carotid bodies are exposed to an elevated systemic blood pressure, and in this case the patient had severe left ventricular hypertension. The carotid body was enlarged, and the area of the largest lobule was considerably greater than the control cases due to the proliferation of type II cells, that were associated with many axons of unknown origin. In this case, type II cells accounted for 73% of the total cells in the carotid body. Type I cells had been compressed and the size of the type I cell clusters was much reduced. Many type I cells were found to have dark staining nuclei with compact chromatin, highly vacuolated cytoplasm clusters had a great degree of type I cell atrophy. Figure reproduced with permission from Heath and Smith (2012)

Although the clinical presentations and underlying pathology of these diseases are very different, excitotoxicity is a common feature that is underpinned by a cascade of neurotoxicity that leads to Ca^2+^ overload, mitochondrial and synaptic dysfunction (Chen et al., 2019; Kumagai et al., 2019), aberrant neuronal transmission and cell death (Armada-Moreira et al., 2020; Iovino et al., 2020) as well as upregulation of proinflammatory processes that exacerbates neuronal pathology (Pascual et al., 2012; Kumagai et al., 2019; Iovino et al., 2020). The carotid body is a potential novel therapeutic target for the treatment of cardiovascular disease. In preclinical models of hypertension and clinically, the carotid body chemoreflex facilitates enhanced sympatho-excitatory responses, that is underpinned by increased excitation of the carotid body characterized by basal tonicity (tonic firing in the absence of a stimulus) and hyperreflexia (enhanced response to a stimulus such as hypoxia); reflecting an obvious similarity to the neural hyperactivity observed in these broad spectrum of CNS diseases. Several other important similarities are also observed in the carotid body associated with metabolic diseases that are analogous to the neuronal changes described in neurodegeneration, including evidence of elevated intracellular Ca^2+^ in glomus cells of hypertensive rats (Weil et al., 1998) (Figure 8), mitochondrial dysfunction that results in increased production of reactive oxygen species and presumably impairs ATP bioavailability (Peng et al., 2003; Li et al., 2007; Ding et al., 2010) as well as increased cellular excitability that results in aberrant neurotransmission (Pijacka et al., 2016; Brognara et al., 2021). These changes are further exacerbated by inflammatory processes including the secretion of cytokines, chemokines, upregulation of their receptors in the carotid body (Del Rio et al., 2012; Fung, 2014) and the subsequent influx of inflammatory cells (Liu et al., 2009a), along with signs of vascular and endothelial dysfunction (Habeck and Holzhausen, 1985; Felix et al., 2012; Schultz and Marcus, 2012). The diverse array of diseases these features are linked to, suggests such pathologies are not specific to an individual cell type or disease, but rather occur because of an imbalance in excitatory and inhibitory mechanisms and reflect ubiquitous pathological traits of dysfunctional signaling. Severe structural and physical changes are also observed in later stages of diseases such as systemic hypertension. For example, in the L-NAME rat model of hypertension, carotid glomus cells were found to display signs of enlargement and cytoplasmic vacuolation while a 31% reduction in the total number of nuclei was observed (Felix et al., 2012), and although an increase in carotid body size is often observed in the adult SHR, this does not necessarily correspond with an increase in the number of type I cells (Atanasova and Lazarov, 2014). In humans, similar features are observed in later stage diseases associated with hypertension including hyperplasia and proliferation of type II cells, akin to astrocyte activation in brain injury (Fix et al., 1996), and an increased percentage in the total number of type I cells per weight of tissue. This is accompanied by an increase in deposition of collagen and extracellular material, a greater density of innervating nerve fibers and the compression of glomus cells, resulting in an increased distance between the regional glomus cell clusters suggesting reduced connectivity of the carotid body cellular network (Heath et al., 1982; Heath and Smith, 2012) and a greater diffusion distance from blood vessels. In addition, glomus cell clusters are hypertrophied, with individual type I cells displaying cytoplasmic vacuolation, dark nuclei and compact chromatin (Heath et al., 1982; Heath and Smith, 2012) indicative of the onset of apoptosis and strikingly similar to the hallmarks of excitotoxicity seen in neurodegenerative diseases (Martin et al., 1998; Elmore, 2007) (Figure 8). Such processes in the carotid body may also occur alongside dementia compounding the issue of autonomic dysfunction in these patients (Allan, 2019). Given the significant overlap in pathological presentation, we propose that there may be more learnings to be extrapolated from our knowledge of the brain and the diseases of the CNS, that may be applied to enhance our understanding the peripheral nervous system including the carotid body.

### ATP-induced glutamate release in the carotid body, or vice versa?

It is known that the electrical excitability of chemoreceptive petrosal neurons is significantly higher in the SHR and in patients with hypertension, and this signal is dependent on type I cell-mediated ATP transmission and activation of P2X2/3 postsynaptic receptors (Pijacka et al., 2016). These purinergic receptors are upregulated and the possibility of altered ATP levels within the carotid-petrosal interface is being investigated (Bardsley et al., 2021). Given the significant overlap between glutamatergic and purinergic signaling in the CNS (Gu and MacDermott, 1997; Shigetomi and Kato, 2004; Rodrigues et al., 2005; Burnstock et al., 2011), it raises the intriguing question as to whether changes in glutamate or GABA signaling within the carotid body may contribute to an imbalance in excitation in hypertension; contributing to (or possibly caused by) altered purinergic signaling. Evidence supporting this hypothesis is found in the CNS, where ATP plays a significant role in facilitating glutamate release, particularly in disease (Burnstock et al., 2011; Foo et al., 2012). Several mechanisms appear to be involved. For example, activation of neuronal P2X7 receptors on presynaptic terminals by ATP has been shown to elicit the release of glutamate or GABA (Llewellyn-Smith and Burnstock, 1998; Sperlágh et al., 2002; Jin et al., 2004; Papp et al., 2004; Wirkner et al., 2005; Sperlagh et al., 2007; Burnstock et al., 2011; Rodrigues et al., 2015; Currò et al., 2020). As such, the release of ATP may have a net excitatory or net inhibitory effect on local circuitry depending on the localisation and subtype of the cell harboring the purinergic receptors (Kato and Shigetomi, 2001; Shigetomi and Kato, 2004; Rodrigues et al., 2015). This idea is pertinent in the context of the carotid body, where ATP has shown to be released from type I cells in response to hypoxia (Buttigieg and Nurse, 2004; Masson et al., 2008). Additionally, it has already been demonstrated, where ATP which exerts excitatory effects on the petrosal ganglion, may in some cases have an inhibitory effect on some type I cells. This inhibitory effect was reportedly via P2Y1 autoreceptor activation but could also presumably be a consequence of GABA_B_ activation, if ATP were to stimulate GABA release from neighboring type I cells for example (Tse et al., 2012). Astrocytes have also been shown to contribute to ATP-induced glutamate release via the activation of ionotropic P2X7 and metabotropic P2Y1 purinergic receptors (Fellin et al., 2006; Malarkey and Parpura, 2008; Pascual et al., 2012) and in some cases Pannexin (Panx-1) channels (Wei et al., 2016). In the carotid body, type II cells (as well as type I cells) are a notable source of extracellular ATP. The activation of type II cell P2Y2 receptors by ATP raises intracellular Ca^2+^ leading to the insertion of Panx1 channels, where ATP diffuses through the pore into the extracellular space, in a process described as ATP-induced ATP release (Zhang et al., 2012; Nurse et al., 2018). Since type II cells also express purinergic receptors, Panx-1 channels, and glutamate transporters, ATP-induced glutamate release is likely in the carotid body. Confirmation of the correlated activity between type I and type II cells by ATP during chemotransduction has been shown in studies using monolayer cultures containing rat type I and type II cell clusters (Nurse and Piskuric, 2013). Depolarisation of type I cells induced by hypoxia, isohydric hypercapnia, or high K^+^ solutions resulted in delayed intracellular Ca^2+^ responses in neighboring type II cells. Considering that type II cells do not respond directly to these specific stimuli (Nurse, 2010; Tse et al., 2012; Zhang et al., 2012), the observed delay is an expected result of cross talk between adjacent type I and type II cells. Evidently, the delayed type II cell responses were also confirmed through the addition of a P2Y2 receptor blocker suramin and the nucleoside hydrolyase apyrase, that prevented the type II cell response indicating endogenous or paracrine release of ATP from neighboring type I cells (Murali and Nurse, 2016).

The relationship between glutamate and ATP release is reciprocal, and significant evidence implicates both players in pathological conditions (Burnstock et al., 2011). In the CNS, prolonged hypoxia and glucose deprivation are known to induce ATP release from neurons and astrocytes which promotes excess glutamate release causing ischaemic damage, neurotoxicity, and neuronal cell death (Choi and Rothman, 1990; Sperlagh et al., 2007; Burnstock et al., 2011). Importantly, glutamate toxicity leading to Ca^2+^ overload and apoptosis has been shown to be directly dependent on the auxiliary role of ATP, where cell death was prevented by administration of P2Y1R antagonists, both *in vivo* and *in vitro* (Simões et al., 2018). Could an increase in ATP release in pathological states stimulate glutamate release from adjacent cells, raising excitation? One alternative source of glutamate within the carotid body may also come from the petrosal ganglion itself (Figure 5). As described, chemoreceptive petrosal afferents are immunoreactive for VGLUT2, suggesting that these sensory neurons may also be a source of glutamate for the carotid body when activated by ATP (Yokoyama et al., 2014), in a manner akin to the sensory neurons of the dorsal root ganglion, that release glutamate in response to purinergic P2X receptor activation (Gu and MacDermott, 1997). Since petrosal afferents and type I and type II cells of the carotid body all express the core components for purinergic and glutamatergic signaling (Figure 5), perhaps a role for ATP-induced glutamate release (or vice versa) in healthy as well as pathological states should be considered.

## Summary

As the primary peripheral chemoreceptor in the body, the carotid body is involved with the maintenance of ventilation and cardiovascular control to ensure adequate perfusion and oxygenation of tissues. However, the carotid body also functions as a multimodal sensor and is sensitive to a plethora of stimuli, that are dynamic and linked to environmental factors. In this manner we might think of the carotid body as a master switchboard for signal integration, where numerous incoming signals are simultaneously received, interpreted, and translated into a range of appropriate physiological outputs. We suggest that at an organ-level, the carotid body morphology is structured in such a way to enable its function as a multifaceted sensor, and propose that each discrete region of clustered cells is both phenotypically distinct and connected to separate lines of communication regulating a range of visceral reflexes and behavioral responses (Figures 1, 3). This is similar in many ways to distinct regions of the brain that can act independently and are characterized by their function, yet are interconnected to other regions permitting parallel processing of information. Intriguingly, the cell types and their roles within the carotid body also share many similarities to the neurons and astrocytes of the brain, including their responses to signals in the form of electrical excitability, vesicular-mediated neurotransmitter release, mechanisms for clearance, and the assistance of a network of supporting cells. Moreover, it appears that the rudimentary components for synaptic plasticity, learning and memory including the presence of glutamate and GABAergic systems and their respective ion-gated and metabotropic receptors are also conserved in the carotid body (Figure 5), where the pathophysiology of common diseases of the carotid body may be linked to deviations in these processes. What the carotid body learns and memorizes might start with set point of reflex sensitivity, for example. Equally, repetitive stimuli such as the one caused by sleep apnoea may result in long term facilitation contributing to aberrant carotid body discharge and sympathetic excess. Many decades of research have contributed to our understanding of the CNS in health and disease, and we suggest that understanding the processes involved in neuronal dysfunction such as in neurodegenerative diseases may be relevant to the carotid body, offering new avenues for therapeutic innovation.

## Author contributions

OG, EB, and JP all conceived the ideas. OG and EB wrote the final draft. OG, JP, AGP, and APP revised and edited the final manuscript. OG and EB drafted the figures. JP raised the funding. All authors revised and approved the final manuscript.
